# Post traumatic growth among police officials who participated in Global war on Terror in FATA (Federally Ministered Areas) Pakistan

**DOI:** 10.12669/pjms.38.3.5324

**Published:** 2022

**Authors:** Azra Azeem, Nelofar Kiran

**Affiliations:** 1 Azra Azeem, National Institute of Psychology, Quaid-e-Azam University, Islamabad, Pakistan; 2 Dr. Nelofar Kiran Rauf, National Institute of Psychology, Quaid-e-Azam University, Islamabad, Pakistan

**Keywords:** Perceived social support, Traumatic growth, Post traumatic stress disorder, Combat

## Abstract

**Background and Objectives:**

Exposure to traumatic event like war evokes certain distress full reactions like post traumatic stress disorder. However, stress is not the only out come as sometimes traumatic event leads towards growth as well. Our objective was to study the relationship between post-traumatic stress, hope, perceived social support and post traumatic growth in police officials who actively participated in war on terror and secondly to highlight the role these factors and demographic variables like marital status and family system play in the promotion of post traumatic growth.

**Methods:**

A cross sectional study was conducted on the officials of police who participated in war on terror and were currently deployed in peaceful areas. Study was planned at Quaid-e-Azam University, Islamabad. Sample of the study comprised of 200 police officials. Sample was collected by using non probability purposive sampling technique. This technique was selected as focus of the study was on particular group of police officials who participated in war on terror and had direct exposure of life-threatening trauma during the war. Scales were used in Urdu language. Perceived social support scale and post traumatic growth inventory were available in Urdu language. Scales that were available in English like PDS-5 (PTSD diagnostic scale) and Adult Hope scale were translated in Urdu language and were culturally adapted. Post traumatic growth inventory was used for measuring post traumatic growth. PTSD diagnostic scale (PDS-5) was used for measuring post-traumatic stress. Perceived social support was measured by using Multidimensional scale of social support and hope was measured by using Adult hope scale.

**Results:**

Age range of the participants was between 21 to 54 years (Mean 33.6, SD= 6.3). Higher exposure to traumatic events was positively related with growth. Study variables like hope and social support showed positive relationship with post traumatic growth. Married and unmarried individuals showed difference on perceived social support (t = 9.31, p = .001), post traumatic growth (t = 8.75, p =.001) and post-traumatic stress (t = 2.04, p = .030). Respondents with extended and nuclear family system showed significant difference on perceived social support (t =2.488, p = .000) and hope (t =2.585, p = .000).

**Conclusion:**

Results of study indicated that PTSD is not the inevitable outcome of combat trauma sometimes trauma leads to post traumatic growth.

## INTRODUCTION

 Global war on terror is basically an international military campaign that was launched by US government after 9/11 terrorist’s attacks. The term originally was used for the countries associated with Al-Qaida.^1^ In war against terror all militant organizations were banned in Pakistan and a campaign was launched in federally administered tribal areas like Waziristan, swat region, sending 80,000 troops to remove Al-Qaida and Taliban forces and several other small groups of terrorists in the area. Pakistan being a front-line state in global war on terror has paid human and other effects much more than any other nation in the world.^1^,^2^ All security agencies played active role and conducted combined operations to remove insurgency from the tribal areas and from the whole country. Pakistani security agencies (Military, Police and Frontier core) for the last almost two decades are facing continued stress and combat environment.^3^ Post-traumatic stress and combat trauma can be the result of post reaction to exposure of tragedies during war and raises the chances of developing mental health problems like PTSD and anxiety disorders.^4^ According to US department of Veterans Affairs National Center for PTSD response to combat trauma is an international medical and nursing issue. As expected, the security agencies individuals who have exposure to trauma could develop symptoms of posttraumatic stress disorder PTSD.^4^,^5^ Much of the treatment studies from trauma literature have focused on the negative adversities that follow traumatic events showing mild to severe impact on the individuals facing it. According to a study conducted on US military during years 2004 and 2009, 20% of Afghanistan and Iraq war veterans had sever anxiety, about 10% are estimated to have PTSD” and “in 2012 about 400,000 war veterans obtained financial assistance for the medical treatment. “As many of today’s veterans and security officials are returning with PTSD, it is important that for their rehabilitation strong understanding of how to treat PTSD effectively as well as to facilitate Posttraumatic Growth in the veterans is the need of the time in order to ensure lasting effect. The Diagnostic and Statistical Manual of Mental disorders (DSM-5) lists PTSD as trauma and stress related disorder which results from exposure to traumatic event (with actual or threatened death).^4^-^6^

 PTSD is not the only outcome of exposure to trauma. Sometimes trauma exposure leads individual towards positive outcome which is labeled as Post traumatic growth. Deriving positive meaning from even adverse experience is a core feature of growth and a precursor to individual positive personal change. The concept of PTG theorizes that some people experience marked improvement when they are exposed to traumatic event or experience trauma.^6^-^8^ The popularity of PTG in the past decade resulted in plethora of research studies and application and incorporation of the above mentioned concept into clinical practices. Combat trauma exposure and stressful environment does not result only in stress it can lead towards growth as well.^7^,^8^

 For psychotherapists it is beneficial to understand PTG along with PTSD and to work with veterans and other population exposed to combat trauma. Literature is present on strong association between combat trauma and development of PTG among individuals exposed to trauma. Research and previous literature have indicated that PTG was associated more with life threatening traumas, injuries and illness then other type of traumas such as traumas related to break up or financial losses. One explanation provided is that such circumstances provide a “wake up” call to the individual and demonstrate the promise of a “second chance”.^8^ The most recent literature on post traumatic growth describes that different variables like Hope and perceived social support increase the possibility of psychological growth in after math of a traumatic event.^9^ Trauma related studies internationally and in Pakistan have suggested that different coping skills and demographic variables like age, marital status and gender can play important role in the rehabilitation of trauma survivors as these factors play role to boost their coping confidence.^6^,^10^,^11^

 In the present study, objective was to see the prevalence of PTSD and PTG in police officials who had actively participated in war against terror and to see the relationship of demographic variables such as marital status and family system with growth. More over relationship among post-traumatic stress, perceived social support, hope and Post traumatic growth was studied to high light the path way of PTG among active police officials of war on terror.

## METHODS

 Ethics committee approval was obtained from National Institute of Psychology, Quaid-e-Azam University Ethics board before beginning the study (D-107-3(128) /PhD). A cross sectional study was conducted on police official who actively participated in global war on terror. Sample comprised of 200 individuals who participated in war against terror in FATA (Federally ministered tribal areas of Pakistan). Non probability purposive sampling technique was used for collection of the data. This sampling technique was used as study had to focus on the particular characteristics of the population that were of interest. Only those individuals were included in the sample that had spent minimum duration of six months in war, had faced life threatening exposure during the war and were now deployed in peace full areas from the last one year. All those participants who had experienced any other trauma like life threatening illness, natural disaster exposure, accident, death of some close family member, were with history of some mental illness of clinical level or with some physical disability were excluded from the sample.

 PDS-5 (Post traumatic stress disorder diagnostic scale), Adult Hope scales were translated in Urdu using the Brisilin method of forward and back ward translation, confirmatory factor analysis (CFA) was done by using Amos. Cronbach Alpha reliabilities were computed for the scales to measure the internal consistency of the items. Alpha reliability of PDS-5 was .89 and for Adult hope scale was .86. These reliabilities showed statistically acceptable level. Available Urdu translated version of Multidimensional perceived social support scale and Post traumatic growth inventory (short farm) were used for assessing perceived social support and post traumatic growth among respondents. Permission from the competent authority was taken before data collection and all individuals were approached individually. Informed written consent was taken from every individual. Information related to identity and address was not collected due to the novelty of the sample.^7^,^12^,^13^

## RESULTS

 A total of 200 participants completed the questionnaire and their data was analyzed using SPSS (Statistical Package for the Social Science) version 21. Response rate was 97%. Analysis included descriptive statistics of socio demographic variables such as education, age, marital status, duration spent in combat area, family system etc. Relationship of study variables were analyzed by computing Pearson correlation. For group comparison as per objectives of the study independent sample t- test was computed using 95% confidence level. Sample age ranged from 21 to 54 years with a mean of (33.6) years, (SD =6.3).

 The mean age of the sample was 33.6 with SD 6.3. There were 121 respondents (61.5%) who were matriculate whereas 50 (25%) were with intermediate level qualification. Seventeen (8.5%) were graduates and with post graduate degree were 10 (5%) respondents. Married and unmarried individuals were 154 (77%) and 46(33%) respectively. One hundred six (52.7%) respondents were with extended family system and 94 (47.3%) with nuclear family system. The mean year of the service of the sample was 12.8 with SD 5.8 (Range 4-23). Sample was representative of officers 9(4.5%) and soldiers and other ranks 191(95.5%). Duration respondents spent in war (Operational) area was 26 (13.0%) one year or more, 46 (23.0) two years or more 128 (64%) three years or more. Details are shown in Table-I.

**Table-I:** Frequency and percentages of demographic variable (N=200).

Variables	F	%
***Designation***		
Officers	9	4.5
Soldiers	191	95.5
***Duration in War area***		
Less than 2 years	26	13.0
2 years or more	46	23.0
3 years or more	128	64.0
***Education***		
Metric	123	61.5
Intermediate	50	25
Graduation	17	8.5
Post graduate	10	5
***Age***		
21 -35	126	63
35 on ward	74	37
***Family type***		
Nuclear	94	47.3
Extended	106	52.7
***Marital status***		
Married	154	77%
Unmarried	46	33%

Table-I shows the distribution of total sample on the basis of their demographic variables such as designation, duration spent in war area, education, age, family type and marital status.

 Relationship of study variables is shown in Table-II. Illustrate the relationship of study variables. Post traumatic stress and post traumatic growth were positively correlated (r = .44). The findings are consistent with previous literature that even in the presence of stress growth can be experience. Post traumatic stress and hope showed significant negative correlation (r = - .73**). Hope showed significant positive correlation (r = .98**) with post traumatic growth. Perceived social support showed significant positive correlation (r = .89**) with PTG and negative correlation (r = -.61**) with Post traumatic stress. Findings are consistent with previous studies.

**Table-II:** Correlation between study variables (N=200).

	Variables	1	2	3	4	Mean	SD
1	PS	-	-.73**	-.61**	. 44	28.1	18.92
2	Hope		-	.75**	.98**	33.63	7.69
3	PSS			-	.89**	28.35	6.26
4	PTG				-	61.60	19.11

* p < .05,

** p < .01

Note: PS= Posttraumatic Stress, Hope = Adult hope, PPS = Perceived social support, PTG = post-traumatic growth.

 Differences between respondents with nuclear family and extended family system on PTG (Post traumatic growth), PTS (Post traumatic stress), PSS (Perceived social support) and hope are shown in Table-III. Individuals with extended family system showed high scores on perceived social support (M= 29.51, SD = 5.21) as compared to nuclear family (M= 27.33, SD = 6.9) and difference was significant (t = 2.488, p = .000). On hope Extended family showed higher scores (M = 32.83, SD = 8.02) and nuclear family showed lower scores (M = 28.79, SD = 6.31) both groups showed significant difference (t = 2.585, p = .000) on independent sample t-test. Nuclear and Extended families respondents showed no significant difference on PTS (Post traumatic stress) and PTG post traumatic growth.

**Table-III:** Differences between individuals from nuclear and extended families on Post traumatic growth, Perceived social support and Hope responses.

Variables	Extended (n = 106)		Nuclear (n = 94)		t	p	95 %CI		Cohen’s d

	M	SD	M	SD			LL	UL	
PTS	30.14	17.46	26.04	20.05	1.460	.064	-1.489	8.975	0.07
PTG	48.93	7.18	34.54	15.73	1.576	.060	-7.66	.425	3.85
Hope	32.83	8.02	28.79	6.31	2.585	.000	-9.23	-.414	0.38
PSS	29.51	5.25	27.33	6.90	2.488	.000	.479	3.88	0.06

***Note:*** CI= Confidence interval, LL=Lower limit; UL =Upper limit.

 Married and unmarried individuals showed significant difference on Post traumatic stress and Post traumatic growth scores and Perceived social support. Table-IV Married individuals showed high level of post traumatic growth (M= 37.00, SD = 6.23) however unmarried individuals showed lesser level of growth (M= 31.08, SD = 7.20). The difference between married and unmarried individuals (t = 8.75, p .001) on independent sample t- test was significant. On posttraumatic stress married individuals showed lesser stress (M = 24.00, SD = 17.45) and unmarried individuals scored relatively high scores (M = 29.84, SD = 15.38) and both groups difference (t = 2.04, p = .030) was significant. Married individuals scored high on perceived social support (M = 41.39, SD = 9.01) then unmarried (M = 31.36, SD = 9.20). Both groups showed significant difference (t = 9.31, p = .000) on independent sample t- test. However, married and unmarried individuals showed no significant difference on hope scores.

**Table-IV:** Differences between married and unmarried individuals 0n Perceived social support, Hope and Post traumatic growth and post traumatic stress responses.

Variables	Married (n = 154)		Unmarried (n = 46)		t	p	95 %CI		Cohen’s d

	M	SD	M	SD			LL	UL	
PSS	41.39	9.01	31.36	9.20	9.31	.000	-17.76	-7.37	0.46
Hope	34.19	9.18	33.46	7.21	.562	.301	-10.033	3.62	0.21
PTG	37.00	6.23	31.08	7.20	8.75	.001	-.8.63	-5.45	0.41
PTS	24.00	17.45	29.84	15.38	2.046	.030	-11.48	-.212	0.07

***Note:*** CI= Confidence interval, LL=Lower limit; UL =Upper limit

## DISCUSSION

 The study provides an initial foray into the role of contributing factors might play in coping with post traumatic stress. Study findings revealed that hope and perceived social support help to cope with Post traumatic stress and contribute to promote growth after traumatic situation.^6^,^12^ Present study showed that one year after exposure to traumatic situation of combat environment police officials showed higher post traumatic growth, hope and perceived social support. Results of the study have highlighted that some factors like perceived social support and hope are the important contributing factors of post traumatic growth. The findings of the study are consistent with previous research as they indicate the importance of the role that contributing factors play in the growth to mitigate the deleterious effects of post traumatic stress. Literature also suggests and support that post traumatic growth influences post traumatic stress in combat survivors.^13^-^15^

 Studies have identified the risk and protective factors of stress among the affected population. Studies have shown that financial problem after the disaster is a risk factor and perceived social support serve as a protective factor.^15^-^17^ In different veteran’s studies community support and mentor ship contains multitude of factors that contribute for growth and boost resilience building among trauma survivors. In the present study identification of the contributing factors of growth suggests the direction of the future research as well as potential areas like educating the exposed population to overcome the traumatic stress.^17^-^19^ Despite the reported negative reactions to tragic events therefore findings of different studies have proved that some people maintain psychological stability and different factors play important role in meaningful positive change. Additionally, hope and social support play buffering role and can affect individual’s capacity to move towards growth even in the presence of stress these results are consistent with previous literature.^17^,^20^-^22^

### Conclusions

In the present study results confirmed that social support and hope play protective role in aftermath of war trauma.

### Limitations of the study

The purposive sampling technique limits the external validity of the findings to other disaster exposed population probability sampling techniques can be the best choice. Another limitation for the study was the use of self-report measures which can be subject to recall biases. Family history of psychopathology was not included which may potentially limit the generalization of the research.

 “For future it is suggested that security agencies like police and military must plan to develop comprehensive soldier mental health fitness program to encourage post traumatic growth among soldiers before deployment in combat area”. For future another suggestion is that after war or any other traumatic event post traumatic mental recovery health interventions must be planned and concept of post traumatic growth may be integrated as a capstone to these interventions especially for exposed population at national level.

### Authors’ Contribution:

**AA:** Designed data collection, statistical analysis & editing of manuscript.

**NK:** Supervised each and every step, reviewed and final approval of the script.
